# Changes in Local Network Activity Approximated by Reverse Spike-Triggered Local Field Potentials Predict the Focus of Attention

**DOI:** 10.1093/texcom/tgaa014

**Published:** 2020-04-24

**Authors:** Abdelrahman Sharafeldin, Vanessa L Mock, Stephen Meisenhelter, Jacqueline R Hembrook-Short, Farran Briggs

**Affiliations:** 1 Department of Neuroscience, University of Rochester School of Medicine, Rochester, NY 14642, USA; 2 Ernest J. Del Monte Institute for Neuroscience, University of Rochester School of Medicine, Rochester, NY 14642, USA; 3 Program in Experimental and Molecular Medicine, Dartmouth College, Hanover, NH 03755, USA; 4 Physiology & Neurobiology Department, Geisel School of Medicine at Dartmouth, Lebanon, NH 03756, USA; 5 Department of Brain and Cognitive Sciences, University of Rochester, Rochester, NY 14627, USA; 6 Center for Visual Science, University of Rochester, Rochester, NY 14627, USA

**Keywords:** attention, cortical layers, local field potential, V1

## Abstract

The effects of visual spatial attention on neuronal firing rates have been well characterized for neurons throughout the visual processing hierarchy. Interestingly, the mechanisms by which attention generates more or fewer spikes in response to a visual stimulus remain unknown. One possibility is that attention boosts the likelihood that synaptic inputs to a neuron result in spikes. We performed a novel analysis to measure local field potentials (LFPs) just prior to spikes, or reverse spike-triggered LFP “wavelets,” for neurons recorded in primary visual cortex (V1) of monkeys performing a contrast change detection task requiring covert shifts in visual spatial attention. We used dimensionality reduction to define LFP wavelet shapes with single numerical values, and we found that LFP wavelet shape changes correlated with changes in neuronal firing rate. We then tested whether a simple classifier could predict monkeys’ focus of attention from LFP wavelet shape. LFP wavelet shapes sampled in discrete windows were predictive of the locus of attention for some neuronal types. These findings suggest that LFP wavelets are a useful proxy for local network activity influencing spike generation, and changes in LFP wavelet shape are predictive of the focus of attention.

## Introduction

The effects of attention on the activity of neurons in the visual cortex have been the subject of much study; however, the neuronal mechanisms that give rise to various attention effects are poorly understood. Perhaps the most straightforward effect of attention is the alteration of neuronal firing rates when subjects attend to visual stimuli overlapping recorded neuronal receptive fields (Moran and Desimone 1985; Motter 1993; Luck et al. 1997). Both facilitation and suppression of neuronal firing rates with attention have been observed (Moran and Desimone 1985; Motter 1993; Luck et al. 1997; Hembrook-Short et al. 2017). We and others have proposed that attentional modulation of neuronal firing rate, whether facilitating or suppressing, depends on the match between neuronal feature selectivity and the features attended in the task (Treue and Martinez-Trujillo 1999; Hembrook-Short et al. 2017). Others have proposed that normalization models explain attentional modulation of neuronal firing rates based on the idea that facilitating attention effectively increases visual stimulus drive, akin to enhancing the contrast of the stimulus (Carrasco et al. 2004; Reynolds and Heeger 2009). All of these proposals are feasible, but none provide an explanation for how attention produces more or fewer spikes in response to a visual stimulus. In other words, the mechanism by which attention alters the biophysics of neurons and/or network properties responsible for generating spikes is still unknown.

An important caveat to most studies of attentional modulation of neuronal activity is that neuronal responses are usually averaged over long timescales. For example, attentional modulation of neuronal firing rate is usually computed over hundreds of milliseconds to several seconds. Computing attentional modulation of neuronal activity over long timescales is inconsistent with evidence suggesting that effects of attention are not constant over these long timescales (Fiebelkorn et al. 2018; Mock et al. 2018). Behavioral and neurophysiological evidence suggests that attention should ramp up as the relevant stimulus change approaches (Ghose and Maunsell 2002). More recent work suggests that attention can wax and wane, even over the course of a single trial lasting a few seconds (Cohen and Maunsell 2011). Unfortunately, there is little data on when attention effects become apparent in individual neurons or whether attention effects appear at different time points within a trial for different neuronal types. Increasing evidence suggests that attention differentially regulates neurons in different cortical layers and belonging to diverse neuronal subclasses (Mitchell et al. 2007; Gregoriou et al. 2012; Snyder et al. 2016; Nandy et al. 2017). Thus, understanding how attention alters neuronal spike generation across neurons located in different cortical layers and belonging to different neuronal classes is necessary for a thorough understanding of the neuronal mechanisms of attention.

Some insight into a possible mechanism for producing more spikes with attention comes from studies of attentional modulation of synaptic efficacy among connected pairs of neurons. In both geniculocortical circuits and local circuits within primary visual cortex (V1), attention significantly enhances the likelihood that a presynaptic input gives rise to a postsynaptic spike (Briggs et al. 2013; Hembrook-Short et al. 2019). Although attention enhances synaptic efficacy for most connected pairs, local V1 circuits transmitting task-relevant feature information are enhanced more than those transmitting task-irrelevant signals (Hembrook-Short et al. 2019). Together, these findings suggest that attention could generate a greater number of spikes in response to a visual stimulus by boosting the efficacy of synapses among circuits conveying task-relevant information. Although the results of these studies were remarkably consistent across connected pairs of neurons, sample sizes of connected pairs of neurons were low due to the inherent challenges of such experiments. Additionally, in order to fully understand the mechanism by which attention alters spike probability in response to a visual stimulus, all of the synaptic inputs onto a single neuron would need to be sampled, posing a significant technical challenge.

In this study, we sought to determine whether local field potentials (LFPs) occurring just prior to spikes and recorded on the same electrode contacts as neuronal spikes could provide information about the local network activity influencing neuronal spike generation. We asked whether the shape of reverse spike-triggered LFPs could predict whether and when attention modulated neuronal spiking. LFPs do not provide the resolution or specificity of intracellular recordings of synaptic inputs to a single neuron. However, because LFPs are thought to reflect both sub- and suprathreshold activities among neurons within hundreds of microns of the recording electrode (Katzner et al. 2009; Buzsaki et al. 2012), it is possible that LFPs include components of local network activity, including synaptic inputs onto neurons within the vicinity of the recording electrode. We devised a novel analysis to capture LFPs just prior to spikes, or reverse spike-triggered LFP “wavelets,” for neurons recorded in V1 of monkeys performing a contrast change detection task requiring covert shifts in visual spatial attention. LFP wavelets were dominated by low-frequency fluctuations, and power spectra generated from LFP wavelets showed prominent peaks around 2–3 Hz. In spite of this common feature, LFP wavelet shapes varied across neurons, but also varied within neurons depending on when their associated spikes occurred during a trial. We performed dimensionality reduction to quantify LFP wavelet shape and measure changes in shape over time and across attention conditions. We confirmed that LFP wavelet shape changes tracked with changes in neuronal firing rate for many neurons in our sample. We then employed a simple classifier to predict monkeys’ focus of attention from LFP wavelet shape averaged over the full 1-s analysis window and from LFP wavelet shape sampled in smaller analysis windows corresponding to different time points in the trial. LFP wavelet shapes sampled in smaller windows were predictive of the locus of attention for some neuronal types. These results suggest that LFP wavelets can serve as a useful proxy for local network activity influencing spike generation. Furthermore, changes in LFP wavelet shape measured at discrete time points were predictive of the focus of attention. Results were also consistent with prior findings showing differential effects of attention among distinct V1 neuronal types (Hembrook-Short et al. 2017).

## Materials and Methods

The data analyzed for this study were collected as a part of previous studies of attentional modulation of single neurons and local field potentials (LFPs) recorded across the cortical layers of primary visual cortex or V1 (Hembrook-Short et al. 2017; Mock et al. 2018; Hembrook-Short et al. 2019; Mock et al. 2019).

Data were collected from 3 monkeys performing a contrast-change detection task requiring shifts in covert visual spatial attention (Hembrook-Short et al. 2017; Mock et al. 2018; Hembrook-Short et al. 2019; Mock et al. 2019). Data from 80 sessions in which monkeys completed at least 1 block of ~ 30 trials per attention condition were used for this study. Attention conditions included attend-toward trials in which monkeys attended toward the visual stimulus overlapping the receptive fields of recorded neurons and attend-away trials in which monkeys attended to an identical stimulus placed equal-distant from the fixation dot and outside the receptive fields of recorded neurons, but within the same hemifield. Of the 80 sessions analyzed, 13 were from Monkey B, 6 were from Monkey O, and 61 were from Monkey E. Sessions from Monkeys B and O involved recordings with single electrodes while those from Monkey E involved recordings with multielectrode arrays. Behavioral performance, reported previously (Hembrook-Short et al. 2017; Mock et al. 2018; Mock et al. 2019), indicated that all 3 monkeys appropriately shifted their focus of covert visual spatial attention according to the fixation dot color cue during these recording sessions.

Single electrodes or multi-electrode arrays were used to record from V1 neurons spanning the cortical layers while monkeys performed the attention task. Single-unit spikes were sorted using the same methods and criteria described previously (Hembrook-Short et al. 2017), or through the following steps: 1) raw voltage signals were filtered with a high cutoff of 8000 Hz using a Bessel filter to generate “wideband” signals, 2) wideband signals were high-pass filtered at 250 Hz using a Butterworth filter to remove low-frequency oscillations, and 3) a threshold set at two times the standard deviation of the mean high-pass filtered wideband signal was used to identify spikes as threshold crossings. Spikes with short interspike-interval violations were removed for spike trains sorted manually and those identified with the threshold crossing method. Just under 2 well isolated neurons were recorded on average per session in which single electrodes were used (Monkeys B and O) and about 6 well isolated neurons were recorded on average during multielectrode recording sessions (Monkey E). Only neurons with at least 100 total spikes across trials of each attention condition were included in these analyses. LFP data were also recorded during all sessions as continuous raw voltage data low-pass filtered at 200 Hz using a Bessel filter and downsampled to 1000 Hz. Spiking and LFP data were analyzed from correctly completed trials of the attention task only. For each trial, spikes and LFPs measured during the last 4 complete grating cycles of the visual stimulus prior to the contrast change were analyzed. Because gratings always drifted at 4 Hz, this analysis window had a duration of 1-s per trial (defined as the full analysis window). Laminar compartment locations of recorded neurons were determined by relative depth of the recording contact/electrode compared to thalamocortical recipient layers, as described previously (Hembrook-Short et al. 2017; Mock et al. 2018; Mock et al. 2019). Neurons were classified as simple or complex cells by computing the f1 to f0 ratio (Skottun et al. 1991) from grating responses measured during attend-away trials.

**Figure 1:**
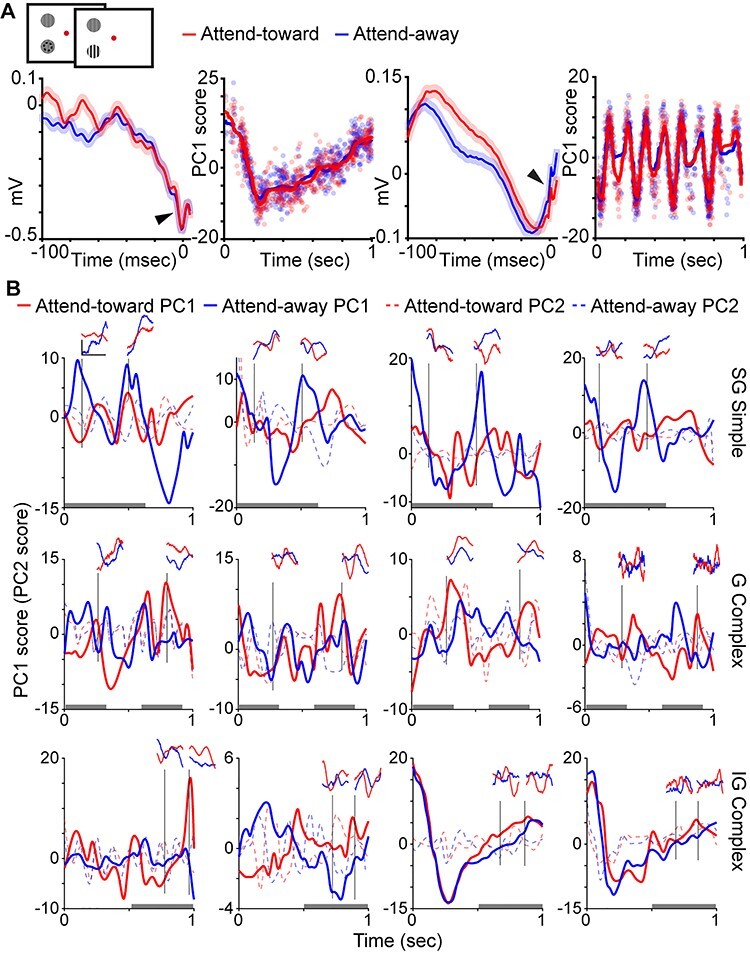
Example of LFP wavelets and time-varying PC scores. *A*. Top: schematic screen shots of the contrast change detection task. Red fixation dot color cued monkeys to attend to the drifting sinusoidal grating inside the receptive field of recorded neurons (dashed circle, not shown in actual task) to detect a change in grating contrast. Bottom: example LFP wavelets for a complex cell (left) and a simple cell (right) both recorded in the granular (G) laminar compartment. Left: curves are average LFP wavelets on attend-toward (red) and attend-away (blue) trials (shading illustrates SEMs). Right: time-varying PC1 scores where dots represent individual LFP wavelet PC1 scores and lines illustrate smoothed time-varying PC1 scores over the duration of an idealized single trial. PC1 scores expressed in arbitrary units. Note neuronal spikes (arrowheads, at time = zero) visible in average LFP wavelets. *B*. Smoothed time-varying PC1 (solid lines) and PC2 (dashed lines) scores for 12 representative neurons with neuronal type and laminar compartment location listed at right (supragranular [SG], granular [G], infragranular [IG]). Shown with each example are 4 individual LFP wavelets (insets) corresponding to specific times during the single idealized trial, delineated by vertical black lines. LFP wavelet scale bar (top left) corresponds to 0.1 mV and 100 msec and applies to all LFP wavelet insets. Red and blue curves illustrate attend-toward and attend-away trial data, respectively. Gray shading at bottom of each plot illustrates the windows over which the classifier correctly distinguished the locus of attention for each neuronal type (Fig. 5).

A set of “LFP wavelets” was computed for each individual neuron as reverse spike-triggered LFPs from spikes recorded on the same contact/electrode. First, LFPs measured during each trial were detrended and then z-scored (Mock et al. 2018). Next, for each spike timestamp, the LFP from 100 msec prior to the spike timestamp through 5 msec following the spike timestamp was extracted to create each LFP wavelet per spike. Then, LFP wavelets were separated by attention condition. Finally, neurons and their LFP wavelets were grouped according to each neurons’ laminar compartment location in the supragranular (SG), granular (G), or infragranular (IG) layers, and by their classification as a simple or complex cell. This grouping yielded 6 classes of neurons/LFP wavelets: SG simple (83 total, 2 from Monkey B, 1 from Monkey O, 80 from Monkey E); SG complex (65 total, 3 from Monkey B, 1 from Monkey O, 61 from Monkey E); G simple (99 total, 5 from Monkey B, 2 from Monkey O, 92 from Monkey E); G complex (49 total, 4 from Monkey B, 2 from Monkey O, 43 from Monkey E); IG simple (83 total, 3 from Monkey B, 1 from Monkey O, 79 from Monkey E); and IG complex (47 total, 6 from Monkey B, 3 from Monkey O, 38 from Monkey E). A small number of neurons (8; not included in the totals above) were excluded because subsequent LFP wavelet analyses generated infinite maximum or minimum values. LFP wavelets varied in shape, but this variation was not different across monkeys: average PC1 scores (see below) were not different across monkeys for neurons in any laminar compartment (*P* > 0.36), and the standard deviations in LFP wavelet shape were not different across monkeys for neurons in any laminar compartment (*P* > 0.08). Accordingly, data from all monkeys were combined. Example average LFP wavelets from attend-toward and attend-away trials for two V1 neurons from different monkeys are illustrated in Figure 1*A*, leftmost panels.

**Figure 2:**
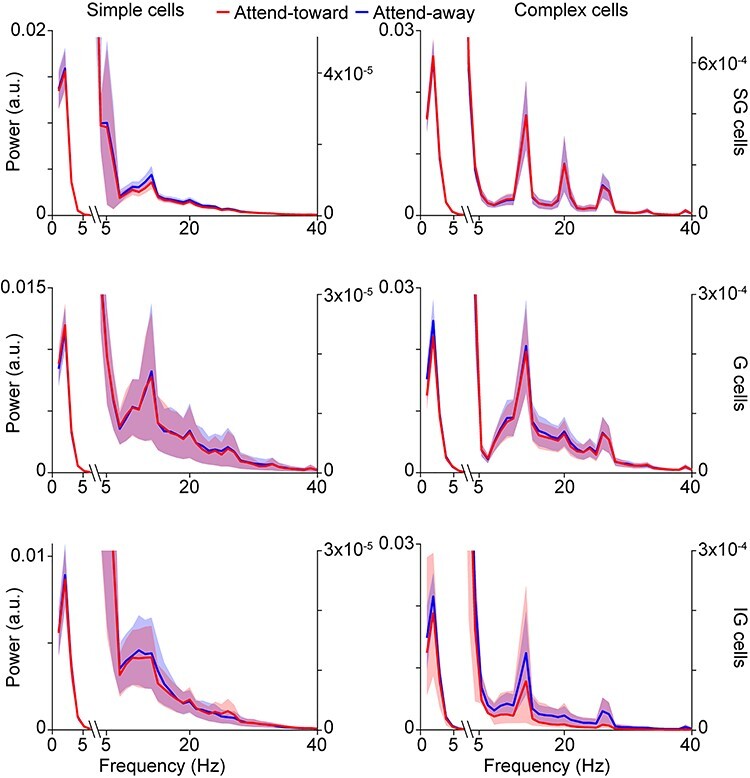
LFP wavelet power spectra**.** Average power spectra for LFP wavelets from neurons of the same type (labeled at top of each column) and laminar compartment location (labeled at right). Red and blue curves correspond to average power spectra for LFP wavelets recorded during attend-toward and attend-away trials, respectively. Shading illustrates SEMs. Note break in *x*-axis and different *y*-axis scales left and right in order to show the large peaks corresponding to low frequencies and much smaller peaks at alpha (~10 Hz) and beta (~20 Hz) band frequencies.

Power spectra were computed for individual LFP wavelets using Welch’s method with a Hamming window of 106 msec and a sampling frequency of 1000 Hz. Power spectra were separated by attention condition and then averaged per attention condition per neuron. Power spectra per attention condition were then averaged across neurons of the same type and laminar compartment location.

**Figure 3:**
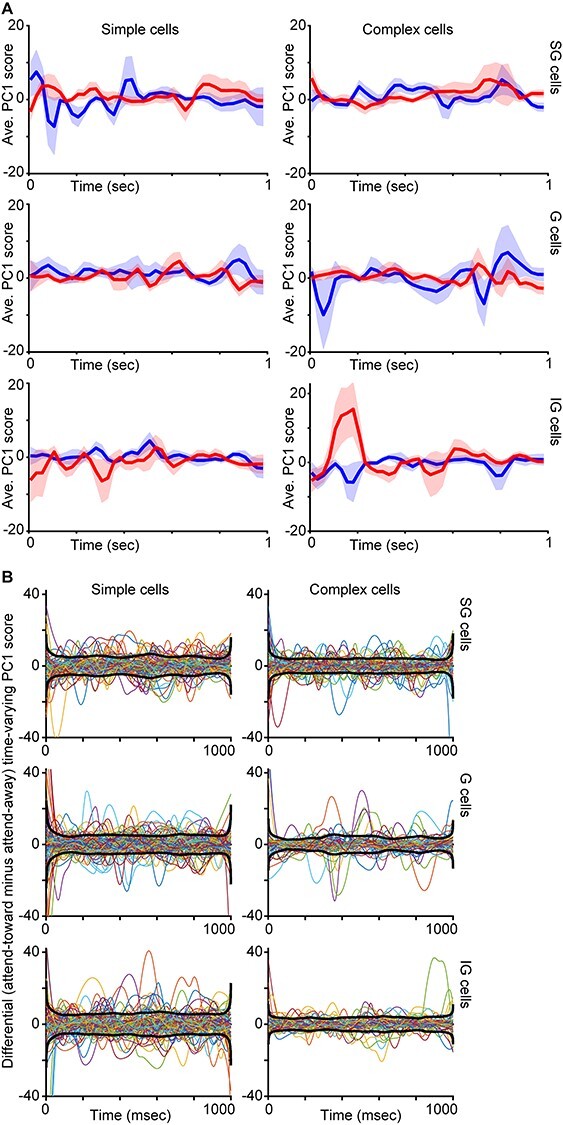
Average time-varying PC1 score curves per neuronal type. *A*. Smoothed time-varying PC1 scores (arbitrary units) averaged across all neurons of the same type and laminar compartment location (labeled as in Fig. 2) with data from attend-toward (red) and attend-away (blue) trials illustrated separately. Shading illustrates SEMs. *B*. Colored curves are spline fits to differential time-varying PC1 scores (attend-toward curve minus attend-away curve, similar to red curves minus blue curves in *A*) per neuron, illustrated separately for neurons of each type and laminar compartment location (labeled as in *A*). Black curves illustrate the average of the envelopes of individual curves.

In order to compute a single value representing the shape of each spike-triggered LFP wavelet, we performed dimensionality reduction on LFP wavelets per neuron using a principal components analysis (PCA). We calculated the first principal component (PC1) and second principal component (PC2) scores for each LFP wavelet. These PC scores provided single numerical representations of the position of each LFP wavelet in the high-dimensional PC space representing LFP wavelet shape. We performed parallel analyses using PC1 and PC2, and we found that PC1 captured more variance in LFP wavelet shape per neuron. Changes in PC2 score over time were more consistent with luminance modulations in the visual stimulus than with attentional modulation (Fig. 1*B*, dashed lines). We therefore used PC1 for all subsequent analyses. To examine possible changes in LFP wavelet shape over time, that is, depending on when their associated spikes occurred during a trial, we first converted all spike timestamps per neuron into spike times relative to the start of each 1-s full analysis window. We then sorted PC1 scores for each LFP wavelet according to their associated spike timestamp within the 1-s full analysis window. This process generated time-varying PC1 scores across a single 1-s window representing a single idealized or aggregate attention trial. Time-varying PC1 scores corresponding to a single, idealized attention trial were computed separately for LFP wavelets occurring on attend-toward and attend-away trials, for each neuron. Examples of time-varying PC1 scores for two representative neurons are illustrated in Figure 1*A*, second and fourth from left. Additional time-varying PC1 and PC2 score curves for 12 neurons are illustrated in Figure 1*B* along with LFP wavelets extracted from specific time points of the idealized single trial. Time-varying PC1 scores were smoothed using a local regression with weighted linear least squares and a second-degree polynomial model. For illustrative purposes only, smoothed, time-varying PC1 score curves were decimated (25-msec bins) and averaged across neurons of the same type and laminar compartment location (Fig. 3*A*). For a separate analysis, time-varying PC1 scores were fit with spline functions and a difference across attention conditions was computed per neuron as the attend-toward curve minus the attend-away curve. These difference curves are plotted for all neurons in Figure 3*B*, along with the average of the envelopes of each curve, again separated by neuronal type and laminar compartment location.

**Figure 4:**
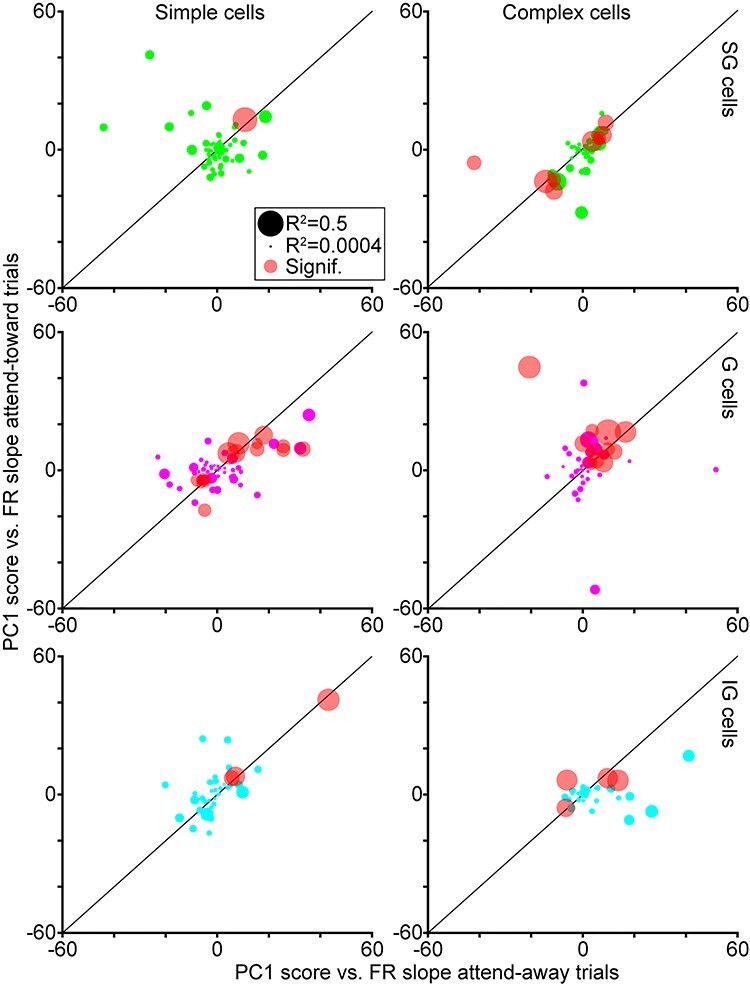
PC1 scores versus neuronal firing rates across attention conditions. Slopes of linear regression fits for comparisons of LFP wavelet PC1 scores and neuronal firing rates on attend-toward and attend-away trials, illustrated separately for neurons of each type and laminar compartment location (labeled as in Fig. 2; SG neurons in green, G neurons in magenta, IG neurons in cyan). Circle size corresponds to the maximum R^2^ for the PC1 score versus firing rate fit. Red circles represent neurons with significant correlations between PC1 scores and firing rates in at least one attention condition.

To test whether PC1 scores correlated with changes in neuronal firing rates, we compared the PC1 score per LFP wavelet with the firing rate in a 100-msec bin preceding each spike and we repeated this for all spikes per neuron. We then computed the correlation between PC1 score and firing rate for each neuron and extracted R^2^, slope, and *P* values for linear regression fits. We performed this PC1 versus firing rate correlation analysis separately for spikes on attend-toward and attend-away trials per neuron. Figure 4 illustrates the slopes and R^2^ values for neurons of each type and laminar compartment, as well as the number of neurons per type for which there was a significant correlation between PC1 score and firing rate in at least one attention condition.

To examine whether LFP wavelets provide information about the locus of attention, we used a simple linear classifier to test whether variations in LFP wavelets for neurons of each type could predict the monkeys’ focus of attention. We performed two separate classification analyses, described below. We evaluated each classification using a receiver operator characteristic (ROC) approach, computing the area under the curve (AUC) for each ROC curve following the assumption that larger ROC AUCs represent better classification of the correct attention trial type (attend-toward or attend-away). For statistical comparisons, we compared ROC curves and AUCs from real data to those computed using shuffled data in which trial type (attend-toward or attend-away) was randomly shuffled.

In the first classification analysis, we tested whether average LFP wavelet shapes on attend-toward and attend-away trials could predict the locus of attention. Average LFP wavelet shapes were defined by their mean LFP wavelet PC1 scores averaged over the full 1-s idealized single trial duration. Input to the classifier was average PC1 scores for all neurons of the same type on attend-toward and attend-away trials. Real and shuffled data were resampled 10 times to provide 10 repeats, and average ROC curves were computed from these repeats. Nonparametric analysis of variance tests with *P* values corrected for multiple comparisons was used to examine significant differences across real versus shuffled data and across neuron types.

In the second classification analysis, we tested whether LFP wavelet shapes sampled in 300-msec windows on attend-toward and attend-away trials could predict the locus of attention. LFP wavelet PC1 scores were sampled every 50-msec within 300-msec windows (sliding the 300-msec window by 100 msec per analysis, for 7 analysis windows across the 1-s trial). Input to the classifier for each window analysis was 6 PC1 scores per neuron for neurons of the same type on attend-toward and attend-away trials. Real and shuffled data were resampled 10 times to provide 10 repeats and average ROC curves were computed per window analysis. Nonparametric analysis of variance tests with *P* values corrected for multiple comparisons was used to examine significant differences across real versus shuffled data for each window and neuron type. Separate significance tests were applied to neuronal populations in each laminar compartment.

**Figure 5:**
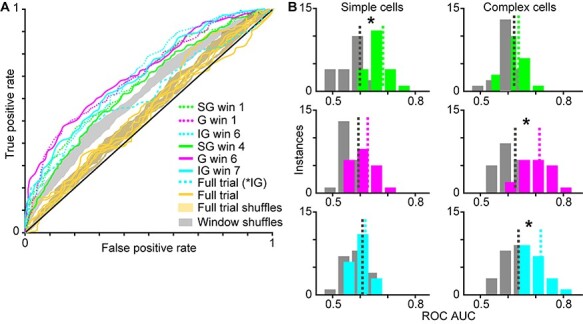
Linear classifier predicts focus of attention for select neuronal types and trial windows. *A*. ROC curves computed from LFP wavelet PC1 scores of all SG simple cells (green), G complex cells (magenta), and IG complex cells (cyan) sampled from earlier (dotted) and later (solid) trial windows (labeled in legend as win 1, etc. with msec ranges listed in Table 1) for which measured ROC AUCs were significantly greater than shuffled ROC AUCs (gray-shaded region illustrates outline of all window shuffled curves; see Table 1 for ROC AUC values). ROC curves computed from LFP wavelet PC1 scores averaged across full trials for each neuronal type and laminar compartment are illustrated in yellow with full trial shuffled curves indicated by yellow shading. Dashed cyan curve illustrates the ROC curve for IG complex PC1 scores averaged across full trials: these data were not different from shuffled data, but this curve was different from the SG simple full trial curve. *B*. Distributions of ROC AUCs for SG (green), G (magenta), and IG (cyan) simple (left column) and complex (right column) cells with shuffled data illustrated in gray. Color-coded dashed lines illustrate distribution means. Only windowed data are illustrated; data from earlier and later trial windows are pooled together in each distribution (shuffled data were computed from the same 2 trial windows per neuron type). Significant differences between real and shuffled ROC AUCs, indicated by asterisks, were observed for SG simple, G complex, and IG complex cells (see Table 1 for statistics per window).

For illustrative purposes, distributions of ROC AUC values from the second classification analysis (the windowing approach) were created for each neuron type to provide a secondary illustration of significant and nonsignificant differences between real and shuffled data for each neuronal type (Fig. 5*B*). ROC AUC values from two windows were pooled to create each distribution. For SG simple cells, G complex cells, and IG complex cells, classification of attention trial type was significantly better with real compared to shuffled data for two windows so ROC AUC values from these two windows were pooled to produce the real and shuffled data distributions. For the other 3 neuronal populations, data from windows 1 and 6 were used to generate real and shuffled ROC AUC value distributions.

## Results

Attention modulates the firing rates of visual cortical neurons; however, the mechanisms that give rise to attention-mediated changes in neuronal spiking probabilities are not known. In order to gain insight into mechanisms underlying neuronal spike rate modulation, we pursued two study objectives. First, we sought a measure of local network activity that could influence spike generation in individual neurons. Second, we asked whether attention alters this local network activity during certain time points within attention task trials in order to adjust neuronal spiking probabilities.

We specifically tested whether reverse spike-triggered local field potentials (LFPs) might serve as a proxy for local network activity influencing neuronal spike generation. Because LFPs contain a mixture of sub- and supra-threshold activity from nearby neurons (Katzner et al. 2009; Buzsaki et al. 2012), LFPs occurring just prior to spikes could contain important information about activity in the local network surrounding a recorded neuron. We extracted reverse spike-triggered LFPs, or LFP wavelets, for spikes from 426 well-isolated single neurons recorded across the cortical layers in V1 of alert monkeys performing a contrast change detection task requiring shifts in covert visual spatial attention. Each LFP wavelet included the LFP 100 msec prior to a spike through 5 msec following the spike. LFP wavelets were always computed from LFPs recorded on the same electrode contact that recorded the neuronal spike train. LFP wavelets for neuronal spikes occurring on attend-toward and attend-away trials of the task were analyzed separately. We separated neurons and their associated LFP wavelets into distinct types based on neuronal simple or complex physiology and supragranular (SG), granular (G), or infragranular (IG) laminar compartment location. Our rationale for this classification was 2-fold. Hubel and Wiesel (1968) first classified V1 neurons as simple or complex based on receptive field structure, and this convention has remained a useful method for distinguishing V1 neurons by thalamic input, receptive field structure, and visual physiological response properties. Furthermore, simple and complex cells are differentially distributed across the cortical layers in V1 (Ringach et al. 2002) and the firing rates of simple and complex cells in distinct cortical layers are also differentially regulated by attention (Hembrook-Short et al. 2017). We elected to use these relatively basic definitions of simple or complex and laminar compartment location to group neurons/LFP wavelets so that results could be directly compared to known physiological and attentional differences across these neuronal types.

LFP wavelets were not flat lines but instead displayed dynamic shapes that usually consisted of broad, that is, low-frequency, deflections (Fig. 1). Indeed, average power spectra for LFP wavelets displayed prominent peaks around 2–3 Hz with much smaller amplitude minor peaks in the alpha and beta frequency ranges (Fig. 2). While there were some differences in the number and amplitude of minor peaks across LFP wavelets from simple and complex neurons located in different laminar compartments of V1, all LFP wavelet power spectra were dominated by low-frequency peaks (Fig. 2). Even though LFP wavelets were dominated by broad, low-frequency deflections, LFP wavelet shapes varied across neurons, across attention conditions, and depending on when their associated spikes occurred within a trial. Figure 1*A* (first and third from left) illustrates average LFP wavelets for 2 example neurons showing variations in wavelet shape across attention conditions. In these 2 examples, the neuronal spike is visible in the average LFP wavelet (Fig. 1*A*, arrowheads). Roughly, a third of the neurons in the dataset had visible spikes at time = 0 of their associated average LFP wavelets. Interestingly, LFP wavelets from the same neuron also varied depending on the time the spike occurred within the trial (Fig. 1*B*, insets). To quantify time-varying shape changes among LFP wavelets, we performed PCA to define each LFP wavelet’s shape with a single numerical value, the PC score. We then aligned the PC scores for all wavelets per neuron to the onset of each trial to generate a time-varying PC score curve over the course of a single idealized attention trial (Fig. 1*A*, second and fourth from left). This analysis was performed separately on LFP wavelets from attend-toward and attend-away trials (Fig. 1*A*, red and blue dots/curves, respectively). Consistent with the fact that LFP wavelets from the same neuron varied depending on the time of the spike within the idealized trial (Fig. 1*B*, insets), PC scores quantifying LFP wavelet shape were dynamic over the course of idealized single trials (Fig. 1*A* and *B*). Interestingly, time-varying LFP wavelet shape changes quantified by scores of the first or second PCs yielded different patterns (Fig. 1*B***,** solid vs. dashed lines). While time-varying PC1 score curves displayed larger amplitude and lower frequency fluctuations over the idealized trial, PC2 score curves often appeared to fluctuate near the frequency of luminance modulations in the visual stimulus (drifting sinusoidal gratings modulating at 4 or 8 Hz). For all neurons in the sample, PC1 captured more variance in LFP wavelet shape than PC2, so all subsequent analyses were performed using PC1 scores.

We next asked whether time-varying PC1 score curves were similar across neurons of the same type and laminar compartment location. Smoothed time-varying PC1 score curves averaged for all neurons of the same type and laminar location revealed some similarities among neurons of the same type and some differences across neuronal types (Fig. 3*A*). While some average time-varying PC1 curves were relatively flat (SG complex, G simple), others displayed differences across attention conditions for discrete windows within the idealized single trial. To assess this further, we computed differential time-varying PC1 score curves by subtracting spline fits for attend-away time-varying PC1 scores from spline fits for attend-toward time-varying PC1 scores per neuron (Fig. 3*B*). As in Figure 3*A*, some of the average envelope curves (black thicker lines in Figure 3*B*) appear qualitatively to be flat (SG complex, G simple, IG simple), whereas the SG simple, G complex, and to a lesser extent IG complex envelope curves appear more undulating. Together, these results provided qualitative evidence that LFP wavelet shape changes were more dynamic with greater potential attentional modulation for some neuronal types (SG simple, G complex, IG complex cells). We followed up these qualitative measures with quantification of LFP wavelet shape changes across neuronal types and attention conditions.

In order to quantify the utility of LFP wavelet shape as a predictor of neuronal activity and attention, we first compared LFP wavelet PC1 scores with neuronal firing rates computed from the same 100-msec time bins. If LFP wavelet shape tracks with neuronal firing rate measured over the same time bin, LFP wavelet shape would be a useful proxy for neuronal network activity influencing spike generation. Another reason to study neuronal activity in such small time bins is the following. While attentional modulation of V1 neuronal firing rates averaged over long (~1 sec) analysis windows is weak (Motter 1993; Luck et al. 1997; McAdams and Reid 2005; Yoshor et al. 2007; Hembrook-Short et al. 2017), neuronal firing rates are not steady over long analysis windows and instead display dynamics over shorter timescales related to visual stimulation and attentional modulation (Hembrook-Short et al. 2017; Mock et al. 2018). When we compared LFP wavelet PC1 scores with neuronal firing rates computed from the same time bins, many neurons displayed correlations between LFP wavelet PC1 scores and firing rates (Fig. 4). The slopes of linear regression fits to PC1 score versus firing rate comparisons were relatively consistent across attend-toward and attend-away trials (Fig. 4). This was especially true for neurons with significant correlations between PC1 scores and firing rate in at least one attention condition (red dots in Fig. 4). Between 2 and 22% of V1 neurons of each type demonstrated significant correlations between PC1 scores and firing rates (Fig. 4; percentage of neurons with significant correlation: SG simple = 2%, G simple = 20%, IG simple = 5%, SG complex = 18%, G complex = 22%, IG complex = 14%). Furthermore, the trend whereby complex cells and G simple cells had larger proportions of neurons with significant PC1 score versus firing rate correlations is reminiscent of the finding that these neuronal types displayed greater attentional facilitation of firing rate (McAdams and Reid 2005; Hembrook-Short et al. 2017). It is also interesting to note that a larger proportion of V1 neurons displayed a significant correlation between PC1 score and firing rate than typically show individually significant attentional modulation of firing rate, which is usually around 10% of recorded neurons (Motter 1993; Luck et al. 1997; Hembrook-Short et al. 2017). Together these findings suggested 2 things: 1) LFP wavelet shape is a good approximation for local network activity influencing spike generation as many V1 neurons demonstrated significant correlations between PC1 score and firing rate, and 2) while PC1 score versus firing rate correlation appeared independent of attention condition, the neuronal types with greater numbers of significant correlations were also those that demonstrated larger attentional modulation of firing rate.

To further quantify possible attention-mediated differences in LFP wavelet shape over a trial, we used a simple linear classifier to test whether LFP wavelet shape was predictive of monkeys’ locus of attention. We performed 2 separate classifications of attention trial type using PC1 scores for neurons of the same type and evaluated each classification using an ROC approach. In the first classification analysis, we tested whether average LFP wavelet shape, computed as the mean LFP wavelet PC1 score over the full idealized single trial analysis window per neuron, could predict the locus of attention, that is, whether a given trial was an attend-toward or attend-away trial. We performed this classification for each neuronal type and compared ROC AUC values for real and shuffled data. There were no differences in ROC AUC values between real and shuffled data for any neuronal type for PC1 scores averaged over the full analysis window (*P* = 0.0117, nonsignificant after multiple comparisons correction; average ROC AUC across all neurons: real = 0.55 ± 0.02, shuffled = 0.54 ± 0.01). Average ROC curves computed from average full trial PC1 scores for each neuronal type are illustrated with yellow curves in Figure 5*A* along with the corresponding shuffled ROC curves (yellow shading). The trend toward significance across the population was driven by one difference across neuronal types that was significant in a two-way comparison: ROC AUC values for IG complex cells were greater than ROC AUC values for SG simple cells (*P* = 0.0012; Fig. 5*A*, IG complex cell curve illustrated in dashed cyan). These results are consistent with prior findings of minimal attentional modulation of individual V1 neurons based on average firing rate computed over long analysis windows (Motter 1993; Luck et al. 1997; McAdams and Reid 2005, Hembrook-Short et al. 2017).

The second classification analysis also tested whether LFP wavelet shape could predict the locus of attention, but this time LFP wavelet shape was quantified from smaller windows within the trial. Specifically, we tested whether binned LFP wavelet PC1 scores within sliding 300-msec windows could predict the locus of attention. We selected a window size of 300 msec because behavioral and physiological evidence suggests that attention fluctuates on this timescale (Fiebelkorn and Kastner 2019), and we previously observed dynamic communication of attention between V1 and the thalamus over the same timescale (Mock et al. 2018). Again, we performed this analysis for each neuronal type and compared ROC AUC values for real and shuffled data. We observed significant differences between real and shuffled ROC AUC values for SG simple, G complex, and IG complex neurons for early and late trial windows (Fig. 5*A*, green, magenta, and cyan dotted and solid lines, shuffled curves outlined in gray; statistics listed in Table 1). Interestingly, these neuronal types had the most undulating envelope curves in Figure 3 and average time-varying PC1 score curves showed attentional deviations in similar windows for which attention trial type could be classified from LFP wavelet shape (Fig. 1*B*). Distributions of real and shuffled ROC AUC values for neurons of each type (Fig. 5*B*) further illustrate the ability of the classifier to correctly discriminate attention trial type based on LFP wavelet shape for SG simple (green), C complex (magenta), and IG complex (cyan) neurons, but not for the other 3 neuronal types.

**Table 1:** Measured and shuffled ROC AUC values per neuronal type, laminar compartment, and trial window. Bold *P* values were below significance threshold after correction for multiple comparisons (*P* < 0.002). Average ROC AUC values represented as mean ± standard error (SE)

	SG Simple	SG Complex	G Simple	G Complex	IG Simple	IG Complex	
**100–300 msec (win 1)**	**0.0010**	0.4274	0.0890	**0.0017**	0.2730	0.4727	*P* value
	0.69 ± 0.01	0.64 ± 0.01	0.62 ± 0.02	0.70 ± 0.02	0.62 ± 0.01	0.66 ± 0.02	Mean ± SE measured
	0.60 ± 0.02	0.63 ± 0.01	0.60 ± 0.01	0.62 ± 0.01	0.61 ± 0.01	0.64 ± 0.01	Mean ± SE shuffled
**200–500 msec (win 2)**	0.0028	0.3075	0.1405	0.0091	0.0036	0.1620	*P* value
	0.68 ± 0.02	0.64 ± 0.02	0.63 ± 0.01	0.71 ± 0.02	0.66 ± 0.01	0.69 ± 0.02	Mean ± SE measured
	0.60 ± 0.01	0.62 ± 0.01	0.60 ± 0.01	0.64 ± 0.01	0.61 ± 0.01	0.64 ± 0.02	Mean ± SE shuffled
**300–600 msec (win 3)**	0.0073	0.3447	0.0073	0.1620	0.0452	0.2123	*P* value
	0.67 ± 0.01	0.65 ± 0.02	0.64 ± 0.01	0.66 ± 0.01	0.65 ± 0.01	0.68 ± 0.02	Mean ± SE measured
	0.61 ± 0.01	0.62 ± 0.02	0.60 ± 0.01	0.63 ± 0.02	0.62 ± 0.01	0.65 ± 0.01	Mean ± SE shuffled
**400–700 msec (win 4)**	**0.0008**	0.3847	0.0257	0.0058	0.0211	0.1212	*P* value
	0.67 ± 0.01	0.64 ± 0.02	0.63 ± 0.01	0.73 ± 0.02	0.66 ± 0.01	0.68 ± 0.02	Mean ± SE measured
	0.60 ± 0.01	0.62 ± 0.02	0.59 ± 0.01	0.63 ± 0.02	0.61 ± 0.02	0.65 ± 0.01	Mean ± SE shuffled
**500–800 msec (win 5)**	0.0113	0.1859	0.0140	0.1405	0.1620	0.0890	*P* value
	0.66 ± 0.01	0.65 ± 0.02	0.64 ± 0.02	0.69 ± 0.02	0.64 ± 0.01	0.68 ± 0.01	Mean ± SE measured
	0.61 ± 0.01	0.62 ± 0.01	0.58 ± 0.01	0.65 ± 0.02	0.62 ± 0.02	0.65 ± 0.01	Mean ± SE shuffled
**600–900 msec (win 6)**	0.6776	0.3075	0.0539	**0.0010**	0.9097	**0.0017**	*P* value
	0.64 ± 0.01	0.64 ± 0.02	0.63 ± 0.02	0.72 ± 0.01	0.61 ± 0.01	0.70 ± 0.01	Mean ± SE measured
	0.63 ± 0.01	0.62 ± 0.01	0.59 ± 0.01	0.63 ± 0.01	0.61 ± 0.01	0.64 ± 0.02	Mean ± SE shuffled
**700–1000 msec (win 7)**	0.2730	0.3447	0.0376	0.0312	0.2730	**0.0004**	*P* value
	0.64 ± 0.01	0.64 ± 0.02	0.64 ± 0.01	0.69 ± 0.01	0.64 ± 0.01	0.74 ± 0.02	Mean ± SE measured
	0.62 ± 0.01	0.63 ± 0.01	0.60 ± 0.01	0.64 ± 0.01	0.61 ± 0.02	0.64 ± 0.01	Mean ± SE shuffled

## Discussion

Although the effects of attention on neuronal spiking activity in the visual cortex have been well described, the mechanism by which more or fewer spikes are produced as a result of directed attention is not known. Prior work demonstrated that attention increases the likelihood that a presynaptic input generates a postsynaptic spike among connected pairs of neurons (Briggs et al. 2013; Hembrook-Short et al. 2019). These findings suggest that attention could adjust local network activity, specifically synaptic inputs onto individual neurons, to modulate spike generation. While measuring synaptic inputs onto individual neurons in behaving monkeys poses a significant technological challenge, we theorized it may be possible to infer local network activity from LFPs recorded on the same electrode as neuronal spikes. We performed a novel analysis, a reverse spike-triggered LFP computation, in order to extract LFP wavelets for each spike per V1 neuron. We used PCA dimensionality reduction to compute PC1 scores as a metric of LFP wavelet shape. We first confirmed that LFP wavelet shapes had unique structure across neurons, and we noted changes in LFP wavelet shape over time on attention trials based on our analysis of time-varying PC1 scores (Figs 1 and 3). Importantly, we established that PC1 scores tracked with changes in neuronal firing rate computed over similar timescales (100-msec bins) and many V1 neurons demonstrated significant correlations between PC1 score and firing rate in at least one attention condition (Fig. 4). Finally, we tested whether LFP wavelet shape was predictive of monkeys’ focus of attention using a simple classifier. We first tested whether LFP wavelet shape averaged over the full 1-s attention trial analysis window was sufficient to predict attention locus and this failed for all neuronal types. However, when we tested LFP wavelet shape sampled in smaller windows from discrete time points across attention trials, LFP wavelet shape was predictive of the locus of attention for SG simple cells and G and IG complex cells (Fig. 5). These results suggest the following: 1) LFP wavelets can serve as a proxy for local network activity influencing spike generation in nearby neurons because LFP wavelet shape changes correlate with firing rate for many V1 neurons, 2) LFP wavelets for select V1 neuronal types can predict the locus of attention when sampled at discrete time points within attention trials, and 3) changes in LFP wavelet shape are most predictive of attention for V1 neuronal types that also demonstrate more robust modulations of firing rate with attention. These findings show that very different approaches can yield consistent attentional modulation, even in V1 where attentional modulations are subtle. They also highlight the power of LFPs in providing a unique perspective on local network activity and gaining insight into the mechanisms underlying attentional modulation of neuronal activity.

We separated V1 neurons into 6 types based on the most general physiological and laminar differences: simple or complex physiology and location within the SG, G, or IG laminar compartments. These simplistic neuronal type categories were chosen based on historical precedent and because prior work illustrated differential attentional modulation of firing rates for an overlapping population of recorded V1 neurons grouped using the same categorization (Hembrook-Short et al. 2017). Specifically, G and IG complex cells displayed the largest facilitation of firing rates with attention, while SG simple cells displayed the most suppression of firing rates with attention (Hembrook-Short et al. 2017). Consistent with these prior results, here we found that G and IG complex cells as well as SG simple cells were best at classifying the locus of attention based on their LFP wavelet shapes defined in discrete windows within attention trials. It is interesting to consider the possibility that LFP wavelet shape changes for G and IG complex cells were indicative of increases in neuronal firing rate with attention while LFP wavelet shape changes for SG simple cells were indicative of decreases in neuronal firing rate with attention. Both increases and decreases in neuronal firing rate could be useful predictors of attention locus from the perspective of downstream decoding neurons, especially if increasing spike rates were characteristic of neurons conveying task-relevant visual stimulus information and decreases in spike rates were indicative of neurons conveying task-irrelevant information.

Results of our LFP wavelet shape versus neuronal firing rate correlation analysis shed some light on this idea. Many G and IG complex cells demonstrated positive correlations between PC1 score, our metric of LFP wavelet shape, and neuronal firing rate (Fig. 4). Additionally, the greatest proportion of neurons in these 2 categories had significant (positive) correlations between PC1 score and firing rate. Positive PC1 versus firing rate correlations for G and IG complex cells suggest that LFP wavelet shape changes correlated with increases in spiking. Furthermore, the fact that the classifier correctly detected the locus of attention based on LFP wavelet shape suggests that LFP wavelet shape changes indicative of increased neuronal firing rate predicted attention directed toward the receptive field of these neurons. Figures 1*B* and 3*A* support this theory in that both illustrate differences in LFP wavelets and time-varying PC1 scores across attention conditions for G and IG complex cells during early and later windows in the trial.

In contrast to the overall picture for G and IG complex cells, SG simple cell data suggest a different relationship between LFP wavelet shape, neuronal firing rate, and attention locus. Unlike G and IG complex cells, many SG simple cells had a negative correlation between PC1 score and firing rate, suggesting that LFP wavelet shape changes indicated a reduction in firing rate among SG simple cells. It is therefore likely that the classifier was able to accurately decode attention locus from SG simple cells’ LFP wavelet shapes because shape changes corresponded to reductions in neuronal firing rate with attention directed toward the receptive field. This notion is supported by Figures 1*B* and 3*A* in which LFP wavelets and time-varying PC1 scores for SG simple cells display smaller amplitude fluctuations on attend-toward trials at the beginning and middle of the trial, which are the same trial windows in which classification of attention trial type based on LFP wavelet shape was successful. Again, these results are consistent with prior findings that SG simple cells in V1 demonstrated the most suppression of neuronal firing rate with attention directed toward the receptive field (Hembrook-Short et al. 2017). These findings also provide additional support for the claim that attentional modulation depends upon the match between neuronal feature tuning and the attended features in the task. In the case of SG simple cells that may be selective for visual stimulus features like color and orientation (Johnson et al. 2008; Garg et al. 2019), their feature selectivity is less relevant for the contrast change detection task leading to a reduction in neuronal activity with attention. Decoding the locus of attention, as well as relevant visual stimulus features, will be aided by both increased activity among neuronal populations encoding task-relevant information as well as decreased activity among neuronal populations encoding task-irrelevant visual information. Together, input from diverse neuronal populations will enhance overall decoding capacity of downstream neurons for visual information. These diverse inputs also enable flexibility when task demands change, that is, when subjects are required to attend to new visual stimulus features.

It is also interesting to consider the time points at which LFP wavelets from SG simple and G and IG complex cells could successfully predict the attention locus. LFP wavelets from SG simple cells were predictive of attention locus during the first and fourth analysis windows, corresponding to early and middle parts of the attention trial. SG simple cells, especially those that are color-selective, could receive direct LGN input and/or local input from parvocellular-recipient neurons in layer 4Cbeta (Johnson et al. 2001; Chatterjee and Callaway 2003), producing shorter-latency responses and earlier integration of visual stimulus information. In contrast, complex cells, especially in the deepest cortical layers have longer response latencies reflecting local circuit inputs (Nowak et al. 1995). Interestingly, LFP wavelets from G complex cells were predictive of attention locus at early and late parts of the attention trial while IC complex cell LFP wavelets were predictive of attention locus only at the latest trial time points. Thus, the time points at which LFP wavelets were predictive of attention for the different neuronal types are consistent with their integration times for incoming visual information. Additionally, accumulation of predictive information about the locus of attention at different time points for different neuronal types could also aid downstream decoding neurons by providing an additional dimension, real time, over which visual and attention signals are available.

Although LFPs are relatively easy to record compared to single units and have become a popular measurement of neuronal activity modulations with attention, interpretation of attentional modulation of LFPs has been challenging in part because the precise nature of the LFP is unknown. Here, we take advantage of the excellent temporal resolution of LFPs and the fact that LFPs are likely to reflect local network activity within hundreds of microns of the recording electrode (Katzner et al. 2009; Buzsaki et al. 2012). By specifically analyzing reverse spike-triggered LFPs, we demonstrate consistent structure in LFP wavelets measured for individual neuronal spikes. This combined with the fact that LFP wavelet shape changes track with neuronal firing rates and can serve as a predictor of attention locus for select neuronal types suggest exciting applications for LFPs in elucidating the neuronal and network mechanisms underlying the effects of attention. In particular, LFP wavelets could be used to track the onset of attention effects with finer temporal resolution compared to conventional methods that rely on spiking rates. Because LFP wavelets are linked to individual neurons, further exploration of neuron-specific attentional modulations with improved temporal precision is feasible. The fact that quite distinct measures of neuronal activity (firing rates averaged over long timescales and LFP wavelet shapes sampled at finer timescales) yield strikingly similar overall effects of attention is encouraging, especially in V1 where attention modulations are subtle. Our results highlight the power of combining neurophysiological measurements to better understand the mechanisms underlying fundamental neuronal processes such as visual spatial attention.

## Notes

We thank Dr. Florian Jaeger and Shraddha Shah for helpful comments on this manuscript.

*Conflict of Interest*: None declared.

## Funding

National Institutes of Health (NEI: EY018683 and EY025219 to F.B. and EY023165 to J.R.H-S.); National Science Foundation (EPSCoR 1632738); the Whitehall Foundation; and the Hitchcock Foundation. V.L.M. was supported by a graduate fellowship from the Albert J. Ryan Foundation. A.S. was supported by a University Research Award from the University of Rochester.
